# Suture or Device? A Real-World Analysis of the Closure Strategies in Patients Undergoing LAA Occlusion

**DOI:** 10.3390/jcm14155245

**Published:** 2025-07-24

**Authors:** Saif Zako, Kathrin Klein, Asena Öz, Maei Elsobki, Philipp Mourikis, Carolin Helten, David Naguib, Malte Kelm, Tobias Zeus, Amin Polzin

**Affiliations:** 1 Department of Cardiology, Pulmonology and Vascular Medicine, University Hospital Düsseldorf, Medical Faculty of the Heinrich Heine University Düsseldorf, 40225 Düsseldorf, Germany; 2 Cardiovascular Research Institute Düsseldorf (CARID), 40225 Düsseldorf, Germany; 3 National Heart and Lung Institute, Imperial College London, London SW3 6LY, UK

**Keywords:** LAA, bleeding, closure device

## Abstract

**Background**: Left atrial appendage occlusion (LAAO) is a valuable alternative to long-term anticoagulation in patients with atrial fibrillation (AF) and a high bleeding risk. However, effective vascular closure following large-bore venous access remains a clinical challenge, particularly in patients with multiple comorbidities. This study compares two venous closure techniques—Z-sutures and the suture-mediated ProGlide™ device—regarding their safety and efficacy in patients undergoing LAAO. **Methods**: We conducted a single-center observational study including 163 patients treated with LAAO between 2021 and 2024. Closure was achieved via a Z-suture (*n* = 126) or the ProGlide™ (*n* = 37) based on operator preference. The primary endpoint was clinically relevant bleeding (BARC ≥ 2). The secondary endpoints included 30-day mortality and hospital stay duration. **Results**: The Z-suture group included older and more comorbid patients. Despite these differences, the bleeding rates were comparable between groups. Clinically relevant bleeding was infrequent (Z-suture: 12.6%; ProGlide™: 13.5%). No 30-day deaths occurred in either group, and their hospital stay durations were similar. **Conclusions**: Both the Z-suture and ProGlide™ techniques demonstrated comparable safety and efficacy. Due to their simplicity and potential cost advantage, Z-sutures may be a practical alternative in routine care for high-risk patients.

## 1. Introduction

Atrial fibrillation (AF) is the most common sustained cardiac arrhythmia worldwide, with a global prevalence affecting over 33 million individuals [1]. It is associated with a significantly increased risk of thromboembolic events, particularly ischemic stroke, with epidemiological studies indicating a fivefold elevated risk compared to that in individuals in sinus rhythm [2]. The majority of thrombi in nonvalvular AF originate from the left atrial appendage (LAA) [3], which has therefore been identified as a primary target for both pharmacologic and non-pharmacologic stroke prevention strategies [4,5]. Traditionally, long-term oral anticoagulation (OAC) with agents such as vitamin K antagonists or direct oral anticoagulants has represented the cornerstone of stroke prevention in patients with AF. However, a substantial proportion of patients—particularly elderly individuals, those with a history of major bleeding, and those with contraindications to anticoagulation—are not suitable candidates for long-term OAC due to their elevated risk of bleeding, adverse drug interactions, and challenges related to adherence and monitoring. In recent years, left atrial appendage occlusion (LAAO) has emerged as a valuable and increasingly utilized non-pharmacologic alternative to long-term OAC in patients with nonvalvular AF who are at a high risk of bleeding complications [5]. By mechanically excluding the LAA from the systemic circulation, LAAO effectively prevents thrombus formation at its most common source, thereby reducing stroke risk without the need for continuous anticoagulation. Despite its therapeutic potential, LAAO, like other minimally invasive cardiac interventions, is accompanied by a distinct profile of procedural risks. One of the most relevant procedural challenges involves vascular access, which carries a well-documented risk of complications, including bleeding, hematoma formation, pseudoaneurysm, and arteriovenous fistula [6]. These access-related complications can, in some cases, necessitate additional medical or surgical intervention and may impact the overall safety and feasibility of the procedure, particularly in high-risk patient populations. Patients undergoing LAA occlusion commonly exhibit an elevated bleeding risk due to factors such as advanced age, a fragile or complex vascular anatomy, and a history of bleeding. The use of large-bore venous sheaths in this setting serves as an additional procedural factor that may increase the likelihood of vascular complications further.

In this context, effective post-procedural hemostasis is critical to minimizing access-site complications, especially in patients with multiple comorbidities. While arterial closure techniques have been extensively studied, data on venous closure systems remain relatively limited, particularly in patients with a history of bleeding. Although randomized data have compared suture-mediated closures (ProGlide™) and figure-of-eight (Z-suture) techniques in LAAO and TEER patients [7], a standardized closure strategy remains elusive. Clinical practice continues to vary based on operator experience, cost considerations, and anatomical factors. Procedurally, the ProGlide™ offers reproducibility and faster times to ambulation but is associated with rare but relevant device malfunctions or suture failures, occasionally requiring reintervention [8]. In contrast, Z-suture closure, while simple and cost-effective, is more operator-dependent and has been linked to a higher risk of local hematoma formation or delayed hemostasis in certain populations [9]. Comparative studies remain limited in LAAO-specific cohorts, reinforcing the need for dedicated evaluations of the closure strategy outcomes in this high-risk group.

Therefore, this study aimed to evaluate the safety and efficacy of two venous closure techniques—the traditional Z-suture and the suture-mediated ProGlide™ device—in patients undergoing LAAO. Our objective was to generate real-world evidence comparing the outcomes between both methods in a high-risk population. We hypothesized that both closure methods would demonstrate comparable safety profiles, with potential economic advantages favoring the Z-suture technique.

## 2. Materials and Methods

**Figure 1:**
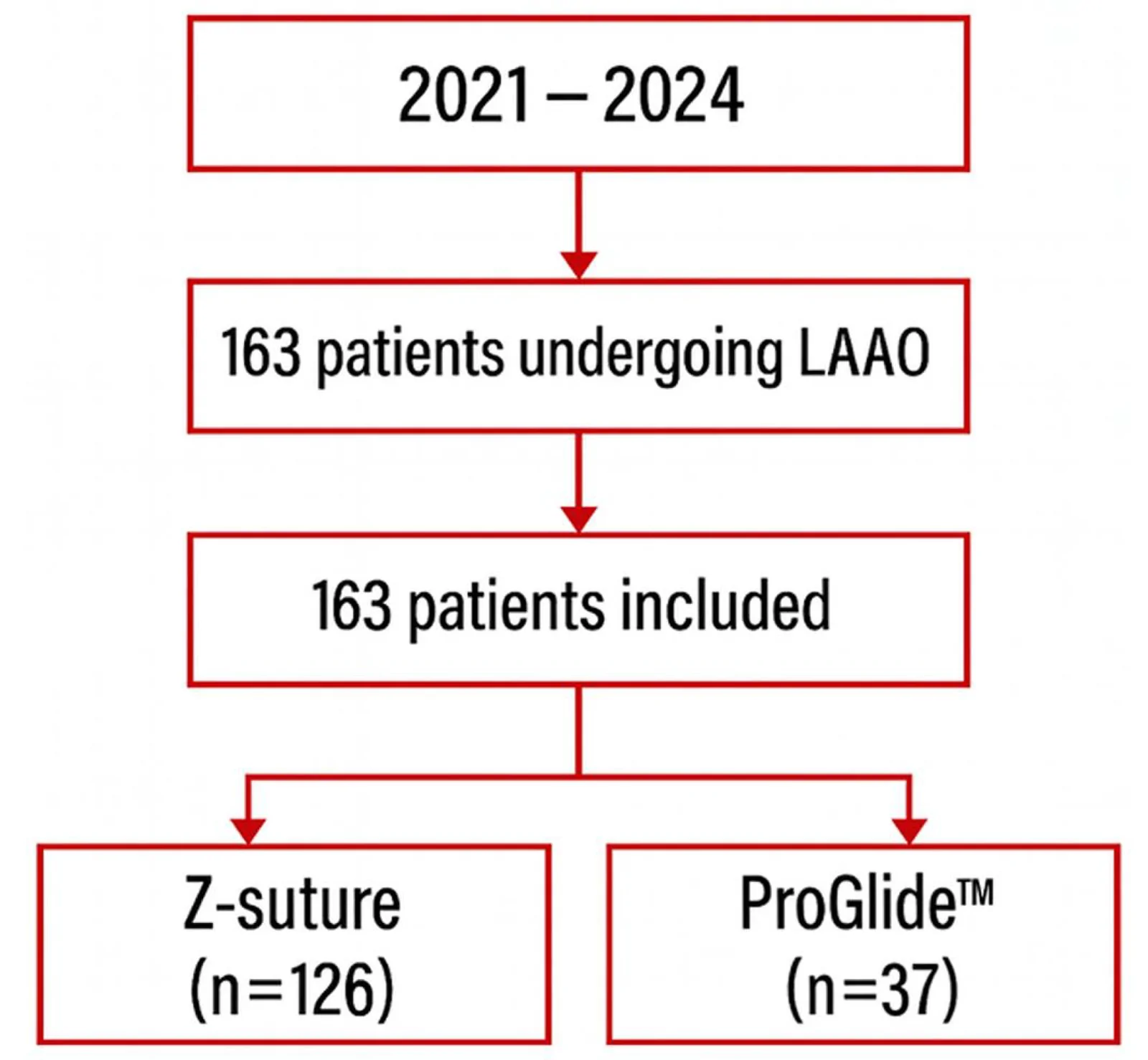
A flowchart depicting the study timeline and procedural grouping. LAAO: left atrial appendage occlusion.

Between 2021 and 2024, this single-center observational cohort study was conducted at the high-volume tertiary care University Hospital Düsseldorf. All patients who underwent left atrial appendage occlusion (LAAO) between 2021 and 2024 were included in this study, resulting in a total of 163 cases. Venous access was established via the femoral vein using a 14 French sheath. Of these patients, 126 received vascular closures via the Z-suture technique and 37 via the Perclose ProGlide™ system (Abbott Vascular Inc., Redwood City, CA, USA), with the closure method selected according to operator preference. Notably, only two primary operators—both highly experienced interventional cardiologists—performed all of the procedures, with one predominantly utilizing the Z-suture technique and the other primarily employing the closure device. The allocation of the closure technique was not randomized but followed a pre-specified assignment based on operator identity. Each of the two interventional cardiologists exclusively applied one closure method throughout the study period—either the Z-suture or the ProGlide™ system—despite both being fully trained and proficient in both techniques. This approach ensured procedural consistency within each technique and minimized intra-operator variability while reflecting the real-world practice conditions at our center. All patients had nonvalvular atrial fibrillation and were considered unsuitable for long-term anticoagulation due to individual clinical considerations. The inclusion criteria comprised symptomatic atrial fibrillation with contraindications to oral anticoagulation (OAC), an age ≥ 18 years, successful percutaneous LAA occlusion, and enrollment in the institutional registry, while the only exclusion criterion was a patient’s refusal to participate in this registry. Venous access was achieved via the femoral veins, and additional arterial access was obtained through the right radial artery for hemodynamic monitoring and contrast injection. The procedure was performed under sedation with the standard hemodynamic monitoring, including ECG, pulse oximetry, and invasive blood pressure. Anticoagulation was maintained using intravenous heparin to achieve a target activated clotting time (ACT) of 250–350 s. All vascular access was made using anatomical landmarks without routine ultrasound guidance. Closure was performed immediately after sheath removal. In the Z-suture group, a figure-of-eight stitch using non-absorbable suture material was applied to the puncture site and secured manually. In the ProGlide™ group, the suture-mediated closure device was applied according to the manufacturer’s instructions. All patients received the standard post-procedural care, including pressure dressing, sandbag application, and four hours of bed rest. Vascular complications were documented as part of routine clinical care by the treating hospital physicians and recorded in the institutional electronic medical record system. Follow-up included regular examination of the venous access site and monitoring of laboratory values, particularly hemoglobin levels. Bleeding events were subsequently reviewed and categorized according to the Bleeding Academic Research Consortium (BARC) classification. Although no independent adjudication committee was involved, all events were classified based on a predefined protocol to ensure consistency and reduce subjectivity. The primary endpoint was the occurrence of clinically relevant bleeding events, defined using the Bleeding Academic Research Consortium (BARC) classification system. Secondary endpoints included all-cause mortality at 30 days and the duration of hospitalization. A visual representation of the inclusion and allocation process is provided in the study flowchart (Figure 1).

### Statistical Analysis

The statistical analysis was performed using SPSS (version 25.0; IBM Corp., Armonk, NY, USA). The normality of the distribution was assessed graphically. Continuous variables were compared using the Student’s *t*-test or the Mann–Whitney U test, as appropriate. Categorical variables were analyzed using the chi-square or Fisher’s exact test. All of the tests were two-sided, and a *p*-value < 0.05 was considered statistically significant. Continuous data are reported as the mean ± standard deviation; categorical variables are given as absolute and relative frequencies. No imputation or propensity score matching was performed.

## 3. Results

A total of 163 patients who underwent left atrial appendage occlusion (LAAO) were included in this study. Of these, 126 patients received venous closures utilizing the Z-suture technique, whereas 37 patients were treated with the ProGlide™ percutaneous suture-mediated closure system. No patients were excluded after initial inclusion due to technical failure, protocol deviation, or incomplete data. All cases meeting the inclusion criteria underwent successful device deployment and were analyzed as part of the final cohort. The baseline demographic and clinical characteristics of both cohorts are detailed in Table 1. The patients in the Z-suture group were significantly older than those in the ProGlide™ group (76 ± 8.5 vs. 71 ± 12.4 years, *p* = 0.04). Diabetes mellitus was markedly more prevalent in the Z-suture cohort (38.1% vs. 14.5%, *p* = 0.008). In addition, the mean CHA_2_DS_2_-VASc score was significantly higher in the Z-suture group (4 ± 1.3 vs. 3.5 ± 3.1, *p* = 0.002). No significant differences were observed between the two groups in terms of gender distribution, body mass index, smoking status, international normalized ratio (INR), left ventricular ejection fraction, or the prevalence of other cardiovascular comorbidities, including arterial hypertension, chronic obstructive pulmonary disease, prior stroke or transient ischemic attack, myocardial infarction, peripheral artery disease, and coronary artery disease. Bleeding outcomes were assessed using the Bleeding Academic Research Consortium (BARC) classification system and are summarized in Table 2. The majority of the patients in both groups did not experience any bleeding events (BARC 0: 60.3% in the Z-suture group vs. 59.2% in the ProGlide™ group; *p* = 0.88). Minor bleeding events (BARC 1) occurred in approximately one quarter of the patients in each group (26.9% vs. 27.0%, *p* = 0.99), with no statistically significant difference. Clinically relevant bleeding events (BARC ≥ 2) were uncommon and similarly distributed across both groups. Specifically, BARC 2 events occurred in 6.3% of the patients in the Z-suture group, compared to 5.4% in the ProGlide™ group (*p* = 0.82). BARC 3a events were reported in 5.6% versus 8.1% of patients, respectively (*p* = 0.70). A single case of BARC 3b bleeding was documented in the Z-suture group (0.7%), whereas no such events were recorded in the ProGlide™ cohort (*p* = 0.47). Importantly, no bleeding events of BARC grade 3c, 4, or 5 were observed in either group. No cases of occluder dislodgement or access-site pseudoaneurysms were observed in the ProGlide group. In the Z-suture group, however, one case of a groin pseudoaneurysm and one arteriovenous fistula were reported—both likely attributable to inadvertent arterial puncture. The mean duration of hospitalization was comparable between the two study groups. Patients treated with the Z-suture technique had an average hospital stay of 3.4 ± 3.73 days, while those who received ProGlide™ closure were hospitalized for an average of 3.59 ± 3.12 days. This difference did not reach statistical significance (*p* = 0.78), indicating that the choice of vascular closure strategy had no measurable impact on the length of post-procedural hospitalization. Within the 30-day follow-up period, no deaths were observed in either group. This indicates a favorable short-term safety profile of both the Z-suture and ProGlide™ closure techniques, with no difference in early mortality between the two strategies.

## 4. Discussion

This study demonstrates that both Z-sutures and the ProGlide™ offer safe and effective closures after large-bore venous access in LAAO. Notably, although the Z-suture group consisted of older patients with a higher burden of comorbidities, the bleeding outcomes were comparable between the two closure strategies. The results of our study align with those of prior investigations into venous closure strategies, including the recent SAFE-VEIN study [10], which compared figure-of-eight suture closures and the Perclose ProGlide™ system. However, it is important to emphasize that the SAFE-VEIN trial included a broader and more heterogeneous patient population, with only approximately 51% of the participants undergoing left atrial appendage occlusion (LAAO). In contrast, our study focused exclusively on a high-risk LAAO cohort. This distinction is clinically significant, as LAAO patients typically present with an advanced age, a greater burden of comorbidities, and a heightened intrinsic risk of bleeding when compared to these values in other structural heart disease populations.

This question of bleeding risk and closure strategy was also addressed in our previous study comparing Z-sutures and the ProGlide™ in a different patient population undergoing patent foramen ovale (PFO) closure [11]. In that younger and generally healthier cohort, both closure techniques proved to be safe and effective, with low complication rates. Building upon those findings, the present study represents the next step by evaluating these closure strategies in a more vulnerable LAAO population characterized by an advanced age, multiple comorbidities, and an inherently higher bleeding risk.

Furthermore, the SAFE-VEIN study also reported no significant differences in 30-day mortality or major bleeding events between the closure techniques, indicating that both suture-based and percutaneous systems offer comparable safety profiles in clinical practice. This observation is consistent with our findings and suggests that although the ProGlide™ is a safe and effective closure option, the traditional Z-suture technique performs equally well in terms of its clinical outcomes—particularly in older, high-risk patients.

Taken together, our current study contributes meaningful real-world evidence, supporting both Z-sutures and the ProGlide™ as viable strategies for large-bore venous closures in the setting of LAAO. Given the comparable safety profiles and absence of a mortality difference, the Z-suture approach may offer a cost-effective and practical alternative in routine clinical practice, especially in resource-conscious healthcare environments.

Importantly, patient characteristics should guide the closure strategy selection. In ambulatory pulmonary vein isolation (PVI), the PRO-PVI observational study demonstrated that the ProGlide™ facilitated immediate hemostasis and safe same-day discharge in 96% of cases, with a mean time to discharge of ~5.8 h and no increase in vascular complications [12]. Conversely, a meta-analysis encompassing nearly 2000 patients showed that figure-of-eight suture closures significantly reduced bleeding (RR 0.30, 95% CI 0.18–0.50) and hematoma formation (RR 0.41, 95% CI 0.25–0.68) compared with that under manual pressure after larger-bore venous access [9]. Together, these findings support a stratified approach—using the ProGlide™ in settings favoring rapid ambulation and applying Z-sutures in high-risk or resource-constrained LAAO cohorts where simplicity and cost effectiveness are key.

Supporting evidence from a small randomized controlled trial comparing figure-of-eight sutures with the Perclose ProGlide in patients undergoing LAAO or TEER found no significant difference in access-site complications or time to hemostasis, with the figure-of-eight approach demonstrating a strong cost advantage (~USD 1 vs. USD 367/device) [7]. Similarly, studies evaluating the ProGlide in pulmonary vein isolation demonstrated faster ambulation without increased complications compared to these findings for Z-suture closures [12], aligning with our data showing that both techniques are clinically safe and effective, though the ProGlide may facilitate earlier mobilization.

Additional insights come from the recently published STYLE-AF study [13], which compared a venous vascular closure system to figure-of-eight suture closures following atrial fibrillation ablation. This randomized, multicenter trial demonstrated that patients treated with a closure device experienced significantly faster hemostasis, earlier mobilization, and shorter hospital stays without an increase in major vascular complications. While these findings highlight the procedural efficiency advantages of device-based closures in the context of AF ablation, our current analysis did not demonstrate a similar reduction in hospital stays in our exclusive LAAO cohort, suggesting that patients’ complexity and comorbidity burden may attenuate some of these benefits in routine practice.

Our study differs from prior trials by exclusively focusing on LAAO patients with a high bleeding risk, a group underrepresented in the existing literature; comparing Z-sutures—a technique often preferred in European centers—to device closures; and providing consecutive real-world data collected from 2021 to 2024, adding pragmatic value beyond controlled RCTs and meta-analyses.

To refine the best practices in this field further, future directions should focus on conducting randomized controlled trials in high-risk populations with uniform baseline characteristics, including stratification by bleeding risk scores such as a HAS-BLED score ≥ 3. The integration of ultrasound-guided vascular access may help minimize inadvertent arterial punctures and reduce bleeding complications. Cost effectiveness analyses comparing suture-based and device-based closure systems across diverse healthcare settings could provide valuable insights. In addition, the evaluation of patient-centered outcomes such as comfort, time to ambulation, and long-term venous patency using vascular ultrasound follow-ups should be prioritized. Future research should also investigate the closure strategies for larger-bore venous access (>16F), where the performance characteristics may differ. In clinical practice, these findings suggest that institutions can select the closure method based on operator experience, procedural efficiency, and economic considerations. The Z-suture technique, with its simplicity and low cost, may be especially beneficial in resource-limited settings, whereas the ProGlide™ may be preferred where reproducibility and time efficiency are critical.

## 5. Limitations

This was a non-randomized, single-center study, and operator selection bias may have influenced the group allocation. Ultrasound-guided punctures were not utilized, which could affect the generalizability to centers employing such techniques; however, given the relatively straightforward anatomy of the femoral venous system, a substantial impact on comparative outcomes is unlikely. Furthermore, the relatively small sample size reduces the statistical power to detect subtle differences, and the observational design implies that unmeasured confounding variables may have impacted the outcomes. Additionally, due to the retrospective nature of our study, data on clinically meaningful secondary endpoints such as time to hemostasis and time to ambulation—frequently reported in previous studies—were not systematically recorded, representing an important limitation. The short 30-day follow-up period also restricts conclusions regarding long-term complications, including venous thrombosis or vessel remodeling. Finally, this study focused exclusively on 14 F sheaths; the outcomes using larger-bore devices remain to be investigated.

## 6. Conclusions

In this real-world cohort of high-risk patients undergoing LAA occlusion, both Z-sutures and the ProGlide™ provided effective venous closure with no significant differences in the complication rates. Z-sutures may offer an economically advantageous alternative for routine clinical practice.

## Figures and Tables

**Table 1:** Baseline characteristics.

	Z-Suture (*n* = 126)	ProGlide (*n* = 37)	*p* Value
Age—years (mean ± SD)	76 ± 8.5	71 ± 12.4	0.04
Gender—male, *n* (%)	88 (69.8%)	24 (64.9%)	0.56
Obesity, *n* (%)	78 (61.9%)	19 (51.4%)	0.25
Weight (kg) (mean ± SD)	85 ± 36	79 ± 18.4	0.35
Height (cm) (mean ± SD)	173 ± 8.9	174 ± 10.1	0.75
BMI (mean ± SD)	26 ± 6.2	25 ± 4.4	0.32
Diabetes mellitus—*n* (%)	48 (38.1%)	5 (14.5%)	**0.008**
Smoking, *n* (%)	30 (24.4%)	6 (16.2%)	0.29
Atrial fibrillation, *n* (%)	126 (100%)	37 (100%)	
CHA2DS2-VASc score (mean ± SD)	4 ± 1.3	3.5 ± 3.1	**0.002**
HAS-BLED score (mean ± SD)	3.4 ± 0.9	3.3 ± 1.3	0.38
INR (mean ± SD)	1.5 ± 2	1.2 ± 0.3	0.35
Ejection fraction (%) (mean ± SD)	54.5 ± 8.0	56.4 ± 14.0	0.47
Arterial hypertension, *n* (%)	120 (95.2%)	34 (91.9%)	0.85
COPD, *n* (%)	27 (21.4%)	4 (10.8%)	0.14
GFR mL/min (mean ± SD)	59.4 ± 23.7	63.0 ± 23.5	0.55
Prior TIA, *n* (%)	6 (4.8%)	1 (2.7%)	0.56
Prior stroke, *n* (%)	33 (26.2%)	10 (27%)	0.91
Prior MI, *n* (%)	17 (13.5%)	5 (13.5%)	0.99
Prior CABG, *n* (%)	13 (10.3%)	1 (2.7%)	0.19
Prior PCI, *n* (%)	43 (34.1%)	15 (40.5%)	0.47
Peripheral artery disease, *n* (%)	21 (16.7%)	5 (13.5%)	0.64
Coronary artery disease, *n* (%)	55 (43.7%)	17 (45.9%)	0.81

**Table 2:** Study outcomes.

BARC Classification	Z-Suture (*n* = 126)	ProGlide (*n* = 37)	*p* Value
BARC 0 (no bleeding), *n* (%)	76 (60.3%)	22 (59.2%)	0.88
BARC 1, *n* (%)	34 (26.9%)	10 (27%)	0.99
BARC 2, *n* (%)	8 (6.3%)	2 (5.4%)	0.82
BARC 3a, *n* (%)	7 (5.6%)	3 (8.1%)	0.70
BARC 3b, *n* (%)	1 (0.7%)	0 (0%)	0.47
BARC 3c, *n* (%)	0 (0%)	0 (0%)	
BARC 4, *n* (%)	0 (0%)	0 (0%)	
BARC 5, *n* (%)	0 (0%)	0 (0%)	

## Data Availability

The data that underpin the findings of this study are fully included in the article. Any additional inquiries or requests for supporting materials can be addressed to the corresponding author.
