# Correction: Zako et al. Suture or Device? A Real-World Analysis of the Closure Strategies in Patients Undergoing LAA Occlusion. *J. Clin. Med.* 2025, *14*, 5245

**DOI:** 10.3390/jcm15197740

**Published:** 2026-10-08

**Authors:** Saif Zako, Kathrin Klein, Asena Öz, Maei Elsobki, Philipp Mourikis, Carolin Helten, David Naguib, Malte Kelm, Tobias Zeus, Amin Polzin

**Affiliations:** 1 Department of Cardiology, Pulmonology and Vascular Medicine, University Hospital Düsseldorf, Medical Faculty of the Heinrich Heine University Düsseldorf, 40225 Düsseldorf, Germany; saif.g.zako@gmail.com (S.Z.); kathrin.klei@web.de (K.K.); asen.oez@gmx.de (A.Ö.); mae.elsobk@gmx.de (M.E.); car.he@gmx.de (C.H.); dav.nagu@gmx.de (D.N.); mal.kel@gmx.de (M.K.); tob.zeu@gmx.de (T.Z.); 2 Cardiovascular Research Institute Düsseldorf (CARID), 40225 Düsseldorf, Germany; 3 National Heart and Lung Institute, Imperial College London, London SW3 6LY, UK

**Reference Update**

In the original publication [1], reference [3] was mistakenly included and is unrelated to the topic of the article. This appears to have happened during the revision process, likely due to a mix-up while updating the reference list. The correct reference intended for citation is as follows:

Zhang, X.; Hou, S.; Liu, W.; Chen, W.; Chen, F.; Ma, W.; Wang, J.; Fan, Y.; Wang, Y.; Chang, D.; et al. Percutaneous Left Atrial Appendage Closure With a Novel LAA Occluder for Stroke Prevention in Atrial Fibrillation. *JACC Asia* **2022**, *2*, 547–556. https://doi.org/10.1016/j.jacasi.2022.04.009. 

With this correction, the order of references was not affected. The authors state that the scientific conclusions are unaffected. This correction was approved by the Academic Editor. The original publication has also been updated.
